# Impact of cow strain and concentrate supplementation on grazing behaviour, milk yield and metabolic state of dairy cows in an organic pasture-based feeding system

**DOI:** 10.1017/S1751731116002639

**Published:** 2016-12-20

**Authors:** C. Heublein, F. Dohme-Meier, K.-H. Südekum, R. M. Bruckmaier, S. Thanner, F. Schori

**Affiliations:** 1 Agroscope, Institute for Livestock Sciences (ILS), Tioleyre 4, 1725 Posieux, Switzerland; 2 Institute of Animal Science, University of Bonn, Endenicher Allee 15, 53115 Bonn, Germany; 3 Veterinary Physiology, Vetsuisse Faculty, University of Berne, Bremgartenstrasse 109a, 3001 Berne, Switzerland

**Keywords:** concentrate supplementation, organic farming, dairy cow, Holstein, pasture

## Abstract

As ruminants are able to digest fibre efficiently and assuming that competition for feed *v*. food use would intensify in the future, cereals and other field crops should primarily be destined to cover the dietary needs of humans and monogastric animals such as poultry and pigs. Farming systems with a reduced or absent concentrate supplementation, as postulated by organic agriculture associations, require adapted dairy cows. The aim of this experiment was to examine the impact of concentrate supplementation on milk production, grazing and rumination behaviour, feed intake, physical activity and blood traits with two Holstein-Friesian cow strains and to conclude the consequences for sustainable and organic farming. The experiment was a cross-over study and took place on an organic farm in Switzerland. In all, 12 Swiss Holstein-Friesian (HCH) cows and 12 New Zealand Holstein-Friesian (HNZ) cows, which were paired according to lactation number, days in milk and age for primiparous cows, were used. All cows grazed full time and were supplemented either with 6 kg/day of a commercial, organic cereal-grain mix or received no supplement. After an adaptation period of 21 days, a measurement period of 7 days followed, where milk yield and composition, pasture dry matter intake estimated with the *n*-alkane double-indicator technique, physical activity based on pedometer measurements, grazing behaviour recorded by automatic jaw movement recorder and blood samples were investigated. Non-supplemented cows had a lower milk yield and supplemented HCH cows produced more milk than supplemented HNZ cows. Grazing time and physical activity were greater for non-supplemented cows. Supplementation had no effect on rumination behaviour, but HNZ cows spent longer ruminating compared with HCH cows. Pasture dry matter intake decreased with the concentrate supplementation. Results of blood analysis did not indicate a strong negative energy balance for either non-supplemented or supplemented cows. Minor differences between cow strains in this short-term study indicated that both cow strains are equally suited for an organic pasture-based production system with no concentrate supplementation. Many factors such as milk yield potential, animal welfare and health, efficiency, grazing behaviour and social aspects influence the decision to supplement grazing dairy cows with concentrates.

## Implications

In the future, competition between feed and food will increase. Ruminants like dairy cows are able to digest forage fibre efficiently. As concentrate supplementation is limited in organic dairy farming, restrictions may cause health problems as energy requirements for high-yielding dairy cows may not be met from forage-only rations. The aim of this study was to verify how supplementation changes the behaviour and production of grazing dairy cows under organic conditions. The two Holstein cow strains investigated in this short-term study are equally suited for an organic, pasture-based farming system with no concentrate supplementation.

## Introduction

In Switzerland, the milk yield per cow has increased steadily over the last years (Bundesamt für Landwirtschaft, 2015), as has the cows’ demand for nutrients and energy. This leads to the question of whether high-yielding dairy cows are still able to meet their energy requirements for efficient milk production on pasture-only diets. Farming systems with a reduced or absent concentrate supplementation, as postulated by organic agriculture associations, require adapted dairy cows. Studies suggest that high producing Holstein-Friesian (HF) cows are less suited for grazing systems, particularly under high stocking rates and seasonal calving systems and without concentrate supplementation (Kolver and Muller, 1998). In New Zealand, HF cows are bred for efficient pasture use with very little concentrate supplementation and seasonal calving (Washburn and Mullen, 2014). They differ in BW, average body condition score (BCS) and milk yield from other HF cow strains (Piccand *et al*., 2013) and have a lower milk production response to concentrate supplementation compared with HF cow strains with a high genetic merit for milk production (Horan *et al*., 2005). In addition, they may be able to use pasture more efficiently for milk production (Macdonald *et al*., 2008) and therefore may be better suited for organic milk production systems. According to Penning and Rutter (2004), behaviour exhibited by animals is an indication of the relationship between their internal state (e.g. nutritional requirements, health) and their environment (e.g. sward state, supplementation and climate). Therefore, grazing behaviour such as grazing duration, herbage intake and intake rate may provide some evidence about well-suited dairy cows and should be examined in detail, as the efficient use of pasture is a priority. According to the knowledge of the authors, this is the first study that investigated the effect of concentrate supplementation on the eating and rumination behaviour, metabolic states and milk production of grazing dairy cows under organic farming conditions.

The aim of this study was to examine the milk yield and milk composition, grazing and rumination behaviour, physical activity and metabolic profile of grazing cows with and without concentrate supplementation under organic conditions. Furthermore, this study aimed to identify differences between two HF cow strains, especially on grazing and rumination behaviour, which could indicate a better suitability for pasture-based milk production systems with restricted concentrate supplementation for organic farming.

## Material and methods

### Animals and experimental design

The experiment was a 2×2 cross-over study with two concentrate levels and two cow strains. All experimental procedures were in accordance with the Swiss guidelines for animal welfare and were approved (no. 2012_10_FR) by the Animal Care Committee of the Canton of Fribourg, Switzerland. Before selecting the cows for the experiment, a medical examination was performed. The experiment consisted of two measurement periods, each consisting of a 21-day adaption period and a 7-day measurement period. For the flow of work and equipment reasons, the cows were equally divided into two consecutive data collection periods of 7 days/measurement period. The experiment took place on the organic farm ‘Ferme École de Sorens’, located 824 m above sea level in Sorens, Switzerland.

A total of 24 HF cows, including 12 Swiss Holstein-Friesian (HCH) cows and 12 HF cows of New Zealand origin (HNZ), were used for the experiment. In all, 14 of them were multiparous and 10 were primiparous. Matched pairs of HCH and HNZ cows were formed according to the number of lactation, days in milk (DIM) and age for primiparous cows. The average economic breeding value (ISEL; Swiss Holstein Breeding Association, Posieux, Switzerland) was 985 (SD 74) for HCH cows. The average economic breeding value for HNZ cows was 806 (SD 70), but this excluded four animals as no breeding value was available.

At the start of the first data collection period, HCH cows had an average number of lactations of 2.3 (SD 1.6), had been 109 (SD 17.9) DIM, had an average BW of 609 (SD 90.1) kg, a BCS of 2.4 (SD 0.28) and were producing 27.6 (SD 3.77) kg milk/day. The HNZ cows had an average number of lactations of 2.7 (SD 2.0), had been 114 (SD 16.7) DIM, had an average BW of 560 (SD 72.3) kg, a BCS of 2.9 (SD 0.24) and were producing 24.1 (SD 5.27) kg milk/day.

### Grazing management, concentrate supplementation and weather conditions

The experiment was carried out in a rotational grazing system from 7 May to 8 July 2012. All 24 experimental cows were managed as a single group separated from the rest of the lactating herd and grazed on pasture from 0800 to 1400 h and from 1800 to 0430 h the following morning. Meanwhile, cows were milked and housed in a free-stall barn. Paddocks were rotationally grazed for 2 to 5 days based on decision rules considering sward height with a reference of 130-mm pre-grazing equivalent to an herbage mass of ~1000 kg dry matter (DM)/ha above 50 mm until a post-grazing sward surface height of 50 mm from ground level. The sward surface height was measured with a pasture meter (C-Dax pasture meter; C-Dax Ltd, Turitea, New Zealand) before cows entered a new paddock and after leaving the paddock. The average pre-grazing sward height was 129 (SD 16.4; *n*=5) mm, corresponding to 1024 (SD 209.3) kg DM/ha above 50 mm in the first measurement period and 122 (SD 11.2; *n*=6) mm, corresponding to 941 (SD 143.8) kg DM/ha for the second measurement period. The average post-grazing sward surface height was 57 (SD 6.6; *n*=5) mm in the first measurement period and 71 (SD 19.2; *n*=6) mm in the second measurement period. Herbage mass above 50 mm (kg DM/ha) was calculated according to: −624.5+12.8×sward height (mm). This regression was calibrated for the pastures of the organic farm ‘Ferme École de Sorens’ (*R* ^2^=0.84; *n*=89). The pastures were long established and composed predominantly of grasses (mainly *Lolium perenne*, *Dactylis glomerata* and *Phleum pratense*), but also of clover (mainly *Trifolium repens*) and herbs (mainly *Taraxacum officinale*). The pastures were fertilized once per year with 25 m^3^/ha of farm-produced manure (corresponding approximately to 80 kg N/ha, 22 kg P/ha and 108 kg K/ha). The chemical composition of the pasture during the measurement periods is presented in Table 1.

**Table 1:** Average chemical composition of concentrate (*n*=2) and pasture (*n*=14) samples (mean±SD).

	Concentrate				Pasture			
Items	Period 1	SD	Period 2	SD	Period 1	SD	Period 2	SD
DM (g/kg of wet weight)	884	1.8	882	1.0	174	30.3	166	20.1
Analysed nutrients and mineral composition (g/kg of DM)								
OM	944	0.3	944	0.2	894	8.6	892	6.2
CP	115	1.3	117	0.3	174	23.0	172	24.5
Ether extract	59	0.1	61	1.2	47	5.3	49	5.0
Starch	502	0.7	504	0.2				
ADF	79	2.2	76	0.7	249	20.8	270	23.4
NDF	221	5.7	228	3.8	397	41.8	427	48.2
Crude fibre	55	1.6	52	0.5	199	25.4	217	23.6
Ca	8.4	0.11	8.7	0.06	8.1	1.40	8.6	2.2
P	6.2	0.03	6.3	0.07	5.1	0.46	5.2	0.54
Mg	3.2	0.01	3.2	0.03	2.1	0.23	2.2	0.24
Na	1.8	0.03	1.8	0.00	0.2	0.05	0.1	0.05
K	7.9	0.05	7.9	0.00	38	3.4	35	1.8
Calculated energy and protein supply^1^ per kg of DM								
NEL (MJ)	8.1	0.02	8.2	0.01	6.3	0.24	6.1	0.32
APDE (g)	79	2.2	76	0.7	104	5.1	102	6.3
Analysed *n*-alkane contents (mg/kg of DM)								
HC32	0.5	0.50	0.7	0.03	5.0	0.66	5.6	1.60
HC33	2.6	0.24	2.5	0.02	59	10.0	65	15.5

DM=dry matter; OM=organic matter; NEL=net energy for lactation; APDE=absorbable protein in the small intestine when rumen fermentable energy is limiting microbial protein synthesis in the rumen; HC32=dotriacontane, C_32_H_66_; HC33=tritriacontane, C_33_H_68_.

1 According to Agroscope (2013).

During an adaptation period of 21 days before both measurement periods, a step-wise provision towards the targeted amount of concentrate (UFA 275 Bio; UFA AG, Herzogenbuchsee, Switzerland), 0 or 6 kg (as-fed basis), took place. The pelleted concentrate was offered to six HCH–HNZ cow pairs in two equal meals (3 kg at 0600 h and 3 kg at 1700 h) after milking in the free-stall barn using separate buckets for each cow. During the measurement period, all 6 kg of concentrate was ingested by all cows with no refusals. The other six HCH–HNZ cow pairs received no concentrate in addition to pasture. Fresh water was always available and a mineral block was available in the barn.

The ambient outdoor temperature was recorded daily by the meteorological station in Grangeneuve (MeteoSchweiz, Station Grangeneuve, Switzerland), located about 15 km north of the experimental pastures. During the first measurement period, the average temperature was 16°C (minimum 13, maximum 19) and 19°C (minimum 15, maximum 24) in the second measurement period.

### Data recording and sample collection

Milk yield (Flo-Master Pro; DeLaval AG, Sursee, Switzerland) was recorded daily and milk composition was analysed from a pooled sample of the morning and evening milk on days 1, 4 and 7 of each data collection period. Milk samples were preserved in tubes containing Broad Spectrum Microtabs II (Gerber Instruments AG, Effretikon, Switzerland) at 8°C. The BW was recorded twice daily after milking and BCS was assessed before each adaptation period and before and after each data collection period according to the five-point system of Edmonson *et al*. (1989).

To estimate individual feed intake on pasture, the *n*-alkane double-indicator technique was used (Mayes *et al*., 1986). Gelatin capsules (HGK 17–60 sl; Capsula GmbH, Ratingen, Germany), containing 0.5 g (weighing accuracy 0.001) alkane marker HC32 (dotriacontane, C_32_H_66_; Argenta Ltd, Auckland, New Zealand) on a carrier of dried fruit pomace, were administered manually with an applicator twice per day starting 6 days before the data collection periods. During the data collection period, a daily spot sample of faeces was taken from each cow with or without stimulus between 0700 and 0800 h. Samples were pooled by cow and collection period and stored at −20°C. Collection of pasture samples started and ended 1 day before the faeces sampling. Pasture sample collection was carried out as described by Graf *et al*. (2005). Daily samples were chopped and stored at −20°C until further analysis. Samples of concentrate were taken daily and pooled per data collection period.

Grazing and rumination behaviour was recorded automatically using a jaw movement recorder with a pressure sensor (Datenlogger MSR145; MSR Electronics GmbH, Hengart, Switzerland; Nydegger *et al*., 2011). The jaw movement frequency and amplitude were measured for 72 h. Data were evaluated with the software programs MSR-Reader (MSR 5.20.01) and MSR-Viewer (Viewer V2), as described in the MSR145 User Manual (MSR Electronics GmbH). The number of grazing and rumination bouts were counted manually from the evaluated output of the software MSR-Viewer. A bout was defined as a sequence of a behaviour not interrupted by any other element of behaviour with a duration >4 min (Metz, 1975) and an inter-bout interval >7.5 min (Dado and Allen, 1994). Grazing bouts contain only those bouts during grazing on pasture not considering bouts when concentrate was eaten in the barn.

Physical activity, including time spent standing, lying and walking was determined using the IceTag^TM^ pedometer (IceRobotics Ltd, Edinburgh, Scotland, UK). The pedometer was attached to the right hind leg of the cow at the metatarsus level and recorded acceleration in three dimensions at 0.1 s intervals for 72 h. Using the software program IceTag-Analyser (V 4.005; IceRobotics Ltd), the data were downloaded and compiled over 60 s intervals. Walking was defined as >3 steps/min, as suggested by Thanner *et al*. (2014).

On days 4 and 5 of each data collection period, blood was collected at 0700 and 1400 h by puncture of the jugular vein, using the Vacuette^®^ System (Greiner Bio-One GmbH,Kremsmünster, Austria). Plasma for the analysis of hormones was retrieved using Vacuette^®^ EDTA tubes (Greiner Bio-One GmbH). After sampling, these tubes were cooled in ice water until they were centrifuged at room temperature (20°C) for 15 min at 3000×**g**. For analyses of blood metabolites and enzymes, Vacuette^®^ serum tubes (Greiner Bio-One GmbH) were stored upside down for 1 h at room temperature (20°C) before centrifugation at 3000×**g** for 15 min and then at 4000×**g** for an additional 5 min (Thanner *et al*., 2014). The retrieved serum and plasma samples were stored at −20°C until they were analysed for hormones, metabolites and enzymes.

### Laboratory analysis

Milk samples were analysed by IR spectrometry (CombiFoss FT+; Foss, Hillerød, Denmark) for contents of fat, protein and lactose (International Dairy Federation, 2000; method number 141C). Urea was determined with a differential pH analyser (Eurochem, Ardea, Italy) before and after hydrolysis with urease (International Dairy Federation, 2004; method number 195). For milk acetone determination, acetone and an internal standard (2-butanone) were transferred via static headspace directly from the milk into the gas phase. The composition of the gas phase was determined with a flame ionization detector on a gas chromatograph (HP 5890 Series II; Agilent Technologies, Santa Clara, CA, USA).

Pasture and faeces samples were lyophilized (Christ, Delta 1-24LSC; Martin Christ Gefriertrocknungsanlagen GmbH, Osterode am Harz, Germany). Concentrate, pasture and faeces samples were milled through a 1.0-mm screen (Brabender mill with titanium blades; Brabender, Duisburg, Germany). Subsamples were dried for 3 h at 105°C to determine DM and subsequently incinerated at 550°C until they reached a stable mass to assess the ash contents. Mineral residues in the ash were dissolved with nitric acid and analysed for Ca, Na, P, Mg and K with inductively coupled plasma optical emission spectrometry (ICP-OES Optima 2000 DV; PerkinElmer, Shelton, CT, USA with system ICP-OES Optima 7300) based on European Standard: EN 155510:2008. The contents of *n*-alkanes HC32 and HC33 (tritriacontane, C_33_H_68_) were determined as described by Peiretti *et al*. (2006). The N content was determined using the Dumas method (Association of Official Analytical Chemists (AOAC), 1995) on a C/N analyser (type FP-2000; Leco Instruments, St. Joseph, MI, USA) and then multiplied by 6.25 to determine the CP content. The ether extract was determined using the Soxtec Avanti 2050 apparatus (Foss, Hillerød, Denmark) for extraction following the guidelines of Verband Deutscher Landwirtschaftlicher Untersuchungs- und Forschungsanstalten 5.1.1. (1993). Acid-detergent fibre (procedure 973.18; AOAC, 1995) was determined with correction for residual ash obtained after incineration at 500°C for 1 h. Crude fibre was analysed in pasture and concentrate (procedure 978.10; AOAC, 1995) and NDF (Mertens, 2002) was assessed with the addition of heat-stable amylase and sodium sulphite. Starch content was determined based on the polarimetric method (method 6493; International Organization for Standardization, 2000).

Metabolite concentrations and enzyme activities were determined using the following commercial test kits: albumin (no. 11970909; Roche Diagnostics, Rotkreuz, Switzerland), alkaline phosphatase (AP, no. 12173107; Roche Diagnostics), alanine aminotransferase (ALAT, no. 63212; bioMérieux, Marcy-l’Etoile, France), aspartate aminotransferase (ASAT, no. 63212; bioMérieux), β-hydroxybutyrate (BHBA; no. RB1007; Randox Laboratories, Crumlin, UK), cholesterol (no. 61218; bioMérieux), creatinine (no. 11489291216; Roche Diagnostics), creatine kinase (CK, no. 61141; bioMérieux), *γ*-glutamyltransferase (GGT, no. 2016788; Roche Diagnostics), glutamate dehydrogenase (GLDH, no. 1929992; Roche Diagnostics), total protein (no. 1553836; Roche Diagnostics), urea (no. 61974, UV 250; bioMérieux), triglyceride (no. 61236; bioMérieux), non-esterified fatty acids (NEFA, no. FA 115; Randox Laboratories) and glucose (no. 1447513; Roche Diagnostics).

Plasma insulin and IGF-1 concentrations were quantified using radioimmunoassay as described by Vicari *et al*. (2008). 3,5,3'-triiodthyronine (T3) and thyroxin (T4) were measured by radioimmunoassay using the Coat-A-Count^®^ Total T3 kit and Coat-A-Count^®^ Total T4 kit, respectively (Siemens Schweiz AG, Zurich, Switzerland).

### Calculations and statistical analyses

Net energy for lactation (NEL) and the absorbable protein in the small intestine when rumen fermentable energy is limiting microbial protein synthesis in the rumen were calculated according to Agroscope (2013). The energy-corrected milk yield (ECM) was calculated based on a 4% fat, 3.2% protein and 4.8% lactose according to Agroscope (2013). Feed intake was calculated using the ratio of the *n*-alkanes HC32 and HC33 on the basis of the equation proposed by Mayes *et al*. (1986).

To double check the intake estimation, the pasture intake without (equation (1), DMI_conc0_) and with concentrate supplementation (equation (2), DMI_conc6_) was additionally calculated according to Baker (2004) based on the recommendations of Agroscope (2013). Changes in BW were not considered, as a period of 1 week is too short to estimate these accurately:1
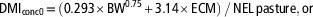

2
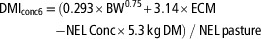
where BW^0.75^ is the metabolic body size (kg BW to the power 0.75), ECM (kg/d) the energy-corrected milk yield, NEL Conc the NEL content of the concentrate (MJ/kg DM) and NEL pasture the NEL content of the pasture (MJ/kg DM).

The statistical analyses were carried out with SYSTAT 13 (Systat Software Inc., Chicago, IL, USA). Data for milk yield and composition, rumination and grazing behaviour, physical activity, feed intake and blood traits were collected over several days and averaged per cow and measurement period. They were analysed using following linear mixed model:
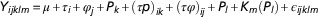
where *Y*_*ijklm*_ is the response, *μ* the least-squares mean, *τ*_*i*_the fixed effect of cow strain *i* (*i*=HCH, HNZ), *φ*_*j*_the fixed effect of the treatment *j* (*j*=conc0, conc6), *P*_*k*_ the fixed effect of the period (*k*=period 1, period 2), (*τp*)_*ik*_ the effect of the interaction between cow strain *i* and period *k*, (*τφ*)_*ij*_ the effect of the interaction between cow strain *i* and treatment *j*, *P*_*l*_the random effect of cow pair *l* (1, … , 12), *K*_*m*_ the random effect of the cow *m* (1, … , 24) and *ε*_*ijklm*_the random error. Models of this type were recommended by Tempelman (2004) with variance components as variance–covariance structure for the repeated measurements. As there were only two periods no alternative variance–covariance structure was envisaged in this study. Not normally distributed data were either logarithmically transformed to fit with normal distribution (NEFA and creatinine kinase) or analysed using R (R Core Team, 2012) with permutation tests for linear models (lactose; Good, 2005). Data presented in the tables were back transformed.

Due to the high proportion of insulin ‘non-detects’ (i.e. values below 3 µU/ml), descriptive statistics was performed as described by Helsel (2012) using R (R Core Team, 2012). Inferential statistics were based on rank methods (Brunner *et al*., 2002). The model was of F1-LD-F2 type (one between-subject factor, two within-subject factors) and ANOVA-type test statistics (Brunner *et al*., 2002) were applied.

The effects were considered significant at *P*⩽0.05. A value of 0.05<*P*<0.10 was considered a trend.

## Results

### Milk yield and milk composition and feed intake

Non-supplemented cows produced less (*P*<0.001) milk and less (*P*<0.001) ECM compared with supplemented cows and HNZ cows had a lower (*P*=0.04) milk yield compared with HCH cows, but no difference between cow strains was observed for ECM (Table 2). An interaction (*P*=0.02) between cow strain and supplementation was reported for milk yield. The HNZ cows produced less (*P*=0.04) milk per kg concentrate than HCH cows.

**Table 2:** Effect of concentrate supplementation and cow strain and their interactions on milk production performance and feed intake

	Conc0		Conc6			*P*-value		
Items	HCH	HNZ	HCH	HNZ	SD	Treatment	Cow strain	Interaction
Milk production performance								
Milk yield (kg/d)	24.9^A^	23.0^B^	30.0^C^	26.0^D^	4.8	<0.001	0.04	0.02
Milk yield concentrate^1^ (kg/kg)			0.84^a^	0.50^b^	0.30		0.04	
ECM^2^ (kg/d)	22.8^A^	22.6^A^	25.3^B^	23.9^B^	3.8	<0.001	0.41	0.16
Fat (%)	3.7^A^	4.0^A^	3.0^B^	3.4^B^	0.6	<0.001	0.08	0.50
Protein (%)	3.2^A^	3.4^B^	3.2^A^	3.5^B^	0.3	0.13	<0.01	0.96
Lactose^3^ (%)	4.4	4.4	4.5	4.5	0.3	0.39	0.50	0.74
Acetone (mg/l)	2.87^A^	2.53^A^	1.52^B^	1.36^B^	0.79	<0.001	0.27	0.68
Urea (mg/l)	239^A^	226^A^	178^B^	176^B^	30.0	<0.001	0.49	0.34
Feed intake								
Pasture DMI (kg DM/day)	12.5^A^	11.7^A^	9.7^B^	9.0^B^	1.7	<0.001	0.09	0.83
Total DMI (kg DM/day)	12.5^A^	11.7^A^	15.0^B^	14.3^B^	1.7	<0.001	0.09	0.83
Calculated pasture DMI^4^ (kg DM/day)	17.3^A^	16.9^A^	12.0^B^	11.0^B^	2.4	<0.001	0.23	0.17
Calculated total DMI^5^ (kg DM/day)	17.3	16.9	17.3	16.3	2.4	0.23	0.23	0.17

Conc0=cows without concentrate supplementation; Conc6=cows with concentrate supplementation; HCH=Swiss Holstein-Friesian; HNZ=New Zealand Holstein-Friesian; DMI=dry matter intake; DM=dry matter. ^A,B,C,D^Means with different superscript letters within the same row differ (*P*<0.01). ^a,b^Means with different superscript letters within the same row differ (*P*<0.05).

1 Milk yield per ingested concentrate (kg/kg).

2 ECM=energy-corrected milk yield (Agroscope, 2013).

3 Log_10_ transformed for statistical analyses.

4 According to Agroscope (2013) and Baker (2004) without BW changes and activity.

5 According to Agroscope (2013) and Baker (2004) without BW changes and activity plus 5.3 kg DM of concentrate.

Milk fat content was greater (*P*<0.001) for non-supplemented cows, but milk protein content was not influenced by supplementation. The HNZ cows had a greater (*P*<0.01) protein content and in tendency a greater milk fat content than HCH cows. No differences were recorded for the lactose content relative to concentrate supplementation or cow strain. The milk contents of acetone and urea of non-supplemented cows were greater (*P*<0.001) compared with supplemented cows, but no differences were observed between cow strains.

For non-supplemented cows, pasture dry matter intake (DMI) estimated with *n*-alkanes was greater (*P*<0.001) than pasture DMI for supplemented cows, but total DMI estimated with alkanes was lower (*P*<0.001) for non-supplemented cows than for supplemented cows. New Zealand Holstein cows tended to have lower pasture DMI and total DMI compared with HCH cows. Calculated pasture DMI based on the requirements was greater (*P*<0.001) for non-supplemented cows, but supplementation had no effect on calculated total DMI and no differences between cow strains were observed for calculated pasture and total DMI.

### Grazing and rumination behaviour and physical activity

Grazing time, mastication (*n*) and mastication rate (*n*/min) were greater (*P*<0.001) for non-supplemented cows compared with supplemented cows and no differences occurred between cow strains (Table 3). The number of grazing bouts was not affected by supplementation, but HNZ cows tended to have greater numbers compared with HCH cows. Non-supplemented cows had a greater (*P*<0.001) duration of eating bouts compared with supplemented cows, but no difference between cow strains was reported.

**Table 3:** Effect of concentrate supplementation and cow strain and their interaction on grazing and rumination behaviour and physical activity over 24 h

	Conc0		Conc6			*P*-value		
Items	HCH	HNZ	HCH	HNZ	SD	Treatment	Cow strain	Interaction
Grazing behaviour over 24 h								
Time (min)	547.1^A^	568.7^A^	442.9^B^	453.0^B^	43.9	<0.001	0.20	0.63
Mastications (*n*)	41 232^A^	42 102^A^	32 070^B^	32 865^B^	3859	<0.001	0.51	0.97
Mastication rate (*n*/min)	75.3^A^	74.0^A^	72.5^B^	72.7^B^	4.0	<0.001	0.73	0.06
Grazing bouts (*n*)	5.2	5.7	5.5	6.0	0.94	0.24	0.10	0.81
Duration of grazing bouts (min)	110^A^	104^A^	81^B^	78^B^	18.8	<0.001	0.42	0.77
Rumination behaviour over 24 h								
Time (min)	381^a^	405^b^	398^a^	423^b^	40.8	0.15	<0.05	0.99
Mastications (*n*)	27 661^a^	29 796^b^	28 888^a^	31 204^b^	3932	0.20	0.04	0.93
Mastication rate (*n*/min)	72.4	73.4	72.7	73.7	5.3	0.47	0.48	0.92
Rumination boli (*n*)	522^a^	578^b^	551^a^	597^b^	69.2	0.22	0.04	0.79
Mastications boli (*n*/boli)	51.5	53.8	54.9	53.4	5.0	0.26	0.83	0.17
Rumination bouts (*n*)	13.1	12.2	13.1	12.7	1.58	0.56	0.14	0.48
Duration of rumination bouts (min)	29	35	31	34	7.0	0.95	0.08	0.40
Activity over 24 h								
Lying (min)	492^A^	541^A^	564^B^	590^B^	62	<0.001	0.05	0.41
Standing+moving (min)	949^A^	900^A^	877^B^	851^B^	62	<0.001	0.05	0.41
Walking (min)	421^A^	419^A^	373^B^	362^B^	59	<0.001	0.75	0.73

Conc0=cows without concentrate supplementation; Conc6=cows with concentrate supplementation; HCH=Swiss Holstein-Friesian; HNZ=New Zealand Holstein-Friesian. ^A,B^Means with different superscript letters within the same row differ (*P*<0.01). ^a,b^Means with different superscript letters within the same row differ (*P*<0.05).

Concentrate supplementation had no effect on traits describing rumination behaviour, but HNZ cows spent more (*P*<0.005) time ruminating, had a greater (*P*<0.005) number of mastication and a greater (*P*<0.005) number of boli compared with HCH cows. No difference between cow strains was observed for mastication rate, mastication per boli and rumination bouts. New Zealand Holstein cows tended to have longer duration of rumination bouts compared with HCH cows.

Non-supplemented cows spent less (*P*<0.001) time lying down, but stood, moved and walked more (*P*<0.001). There was a trend recorded for HNZ to stand and move less and lie down more, but no difference was observed for walking. No significant interaction of concentrate supplementation and cow strain was observed.

### Blood traits

Non-supplemented cows had lower (*P*<0.01) serum glucose concentration but greater (*P*<0.001) concentration of serum BHBA, NEFA and urea compared with supplemented cows (Table 4). Concentration of total protein in serum indicated a tendency to be affected by supplementation, with a greater concentration for non-supplemented cows. Concentrate supplementation had no effect on concentration of serum albumin, triglycerides, cholesterol and creatinine. The activity of creatinine kinase, GLDH and GGT was not affected by concentrate supplementation, but activity of ASAT and ALAT were greater (*P*=0.04) and activity of AP was lower (*P*<0.001) for non-supplemented cows.

**Table 4:** Effect of concentrate supplementation and cow strain and their interaction on blood metabolites, enzymes and hormones

	Conc0		Conc6			*P-*value		
Items	HCH	HNZ	HCH	HNZ	SD	Treatment	Cow strain	Interaction
Glucose (mmol/l)	3.15^A^	3.31^B^	3.25^C^	3.46^D^	0.18	<0.01	<0.01	0.54
BHBA (mmol/l)	0.91^A^	0.82^A^	0.68^B^	0.69^B^	0.17	<0.001	0.45	0.30
NEFA^1^ (mmol/l)	0.12^A^	0.14^A^	0.08^B^	0.09^B^	0.05	<0.001	0.48	0.62
Urea (mmol/l)	4.86^A^	4.77^A^	3.68^B^	3.73^B^	0.90	<0.001	0.95	0.72
Total protein (g/l)	73.6	72.9	72.0	72.2	4.20	0.10	0.88	0.48
Albumin (g/l)	38.8	39.2	38.0	38.9	2.40	0.27	0.32	0.56
Triglycerides (mmol/l)	0.30	0.33	0.30	0.31	0.08	0.44	0.28	0.74
Cholesterol (mmol/l)	6.28	6.26	6.08	6.43	1.10	0.94	0.70	0.24
Creatinine (µmol/l)	73.8	70.7	76.2	69.5	8.2	0.66	0.07	0.19
Creatine kinase^1^ (U/l)	153	191	146	176	55.2	0.26	0.13	0.79
GLDH (U/l)	14.4	16.1	13.1	16.7	4.11	0.71	0.08	0.34
GGT (U/l)	22.8	24.7	22.8	24.8	4.60	0.90	0.20	0.97
ASAT (U/l)	71.2^a^	74.8^a^	67.6^b^	72.8^b^	7.38	0.04	0.15	0.52
AP (U/l)	39.1^A^	50.6^A^	48.9^B^	61.2^B^	21.20	<0.001	0.17	0.86
ALAT (U/l)	29.2^A^	31.7^B^	26.6^C^	29.5^D^	2.60	<0.001	<0.01	0.65
IGF-1 (ng/ml)	91^a^	109^b^	113^c^	141^d^	31.5	<0.001	<0.05	0.25
T3 (nmol/l)	2.28^A^	2.36^A^	2.45^B^	2.60^B^	0.31	<0.001	0.18	0.34
T4 (nmol/l)	49.0^a^	48.7^a^	51.6^b^	53.5^b^	12.01	0.04	0.84	0.51
Insulin (µU/ml)	4.31^a^	3.81^a^	5.82^b^	5.92^b^	3.54	0.03	0.94	0.58

Conc0=cows without concentrate supplementation; Conc6=cows with concentrate supplementation; HCH=Swiss Holstein-Friesian; HNZ=New Zealand Holstein-Friesian; NEFA=non-esterified fatty acids; GLDH=glutamate dehydrogenase; GGT=*γ*-glutamyltransferase; ASAT=aspartate aminotransferase; AP=alkaline phosphatase; ALAT=alanine aminotransferase; T3=3,5,3'-triiodthyronine; T4=thyroxin. ^A,B,C,D^Means with different superscript letters within the same row differ (*P*<0.01). ^a,b,c,d^Means with different superscript letters within the same row differ (*P*<0.05).

1 Log_10_ transformed for statistical analyses.

Concentrate supplementation had an impact on the plasma concentration of the hormones T3 and T4 with lower (*P*<0.001 and *P*=0.04, respectively) concentrations for non-supplemented cows. Non-supplemented cows had a lower (*P*=0.03) plasma insulin and lower (*P*<0.001) IGF-1 concentration compared with supplemented cows.

Differences between cow strains were recorded for serum glucose concentration with lower (*P*<0.01) concentration for HCH cows. Furthermore, HCH cows had a lower (*P*<0.01) activity of ALAT and a lower (*P*<0.05) concentration of IGF-1. For other blood traits no differences between cow strains were recorded. No interactions of concentrate supplementation and cow strain for blood traits were observed.

## Discussion

Many studies investigated the effects of concentrate supplementation on intake, milk production, body condition, grazing behaviour and digestion under non-organic farming conditions, reviewed by Bargo *et al*. (2003) for grazing dairy cows. Studies under organic farming conditions focussed more on the effects on health and fertility on farm level without considering the basic effects of concentrate supplementation (Sehested *et al*., 2003; Ertl *et al*., 2014; Ivemeyer *et al*., 2014). According to the knowledge of the authors no other study investigated the effect of concentrate supplementation on the eating and rumination behaviour, intake, metabolic states and milk production of grazing dairy cows under organic farming conditions. Changes in organic herbage quality, as found by Spann *et al*. (2007), might be partly due to the principles of organic agriculture (International Federation of Organic Agriculture Movements, 2015), for instance, prohibition of synthesized fertilizers and pesticides, as well as with the general promotion of natural, multispecies pastures. As the nutritive value and the fibre content could influence the outcome of concentrate supplementation on grazing dairy cows, studies like ours are needed to close the gap. Finally, only few studies investigated the grazing and rumination behaviour, especially bites per rumination bolus, when investigating the effects of concentrate supplementation of grazing dairy cows.

### Effect of concentrate and consequences for pasture-based organic farming

In accordance with Bargo *et al*. (2003), milk yield and ECM increased for supplemented cows, but to a smaller extent. The observed interaction between concentrate supplementation and cow strain for milk production indicate the different genetic potential for milk production between the two cow strains (Bargo *et al*., 2002; Horan *et al*., 2005). Supplemented HCH cows responded to the extra energy supply with greater milk yield compared with HNZ cows, but ECM was similar.

The reduced milk fat content of supplemented cows is in agreement with other studies, when cows received >4 kg/day of concentrate (Bargo *et al*., 2002; Horan *et al*., 2005). Furthermore, the increased activity of plasma AP in supplemented cows may indicate acidotic stress. To ensure rumen health, rumination is a key factor as it influences salivation and therefore rumen buffering. In the current study, concentrate supplementation had no effect on rumination behaviour. It would have been expected that with increasing pasture DMI, time ruminating, rumination mastication and number of boli increased (Beauchemin and Rode, 1997; McCarthy *et al*., 2007b). The absence of differences in rumination mastication per bolus and number of boli may indicate a similar ease of bolus formation and swallowing for both supplemented and non-supplemented cows, although higher grain diets reflect a greater ease of bolus formation (Beauchemin and Rode, 1997). Rumination mastication per bolus was in the normal range. This poses the question of whether characteristics of rumination behaviour are a suitable indicator of sufficient fibre supply and therefore rumen health, at least in grazing dairy cows.

In agreement with other studies, supplementation caused a substantial reduction in grazing time (Bargo *et al*., 2002; McCarthy *et al*., 2007b) and therefore reduced grazing mastication and grazing mastication rate. This implies the lower motivation of supplemented cows to graze and is supported by the reduced duration of grazing bouts for supplemented cows, as grazing time or grazing time coupled with intake rate (bite rate and size) at the same BW might be indicators for the feeding drive (McCarthy *et al*., 2007b; Prendiville *et al*., 2010).

In accordance with other studies, pasture DMI decreased with concentrate supplementation, which is expressed as substitution rate (Bargo *et al*., 2002; McCarthy *et al*., 2007b). Both calculated and estimated (using *n*-alkanes) pasture DMI were lowered by concentrate supplementation. In contrast, total DMI of supplemented cows was greater or at least the same as the total DMI of non-supplemented cows. However, with reduced pasture DMI for supplemented cows, cheap pasture is substituted by expensive concentrate which is an economic aspect for the farmer. Furthermore, importing ingredients of a commercial organic concentrate mix disturbs the holistic approach of organic guidelines. In the current study, the energy:protein ratio may be balanced as concentrations of urea in blood and milk were in the normal range, with elevated values for non-supplemented cows. In organic farming, the risk of excessive protein intake from pasture might be reduced as the CP content of organic pasture is lower compared with conventional pasture (Spann *et al*., 2007).

Current results indicate a strong relationship between physical activity and grazing behaviour. As supplemented cows spent less time grazing, they spent less time standing and walking. In a pasture-based feeding system, where energy might be the first limiting nutrient, physical activity on pasture is an important factor to be considered. Grazing cows have a greater energy expenditure compared with cows fed indoors, as grazing cows take more steps, spend less time lying down and spend more time eating (Kaufmann *et al*., 2011). According to the Commonwealth Scientific and Industrial Research Organisation (2007), energy requirements for maintenance may increase in the range of 10% to 50% depending on grazing conditions, digestibility of pasture, distance walked, weather, topography and interactions between these factors. Thus, supplemented cows on pasture did not only ingest more energy, but also presented improved energy balance due to energy savings in relation to shorter grazing and physical activities.

A lack of energy in early lactation can cause metabolic problems such as ketosis. In the current study, the increased acetone concentration in milk for non-supplemented cows indicates a small risk of ketosis. This is confirmed by an increased concentration of BHBA and NEFA and decreased glucose and insulin concentration for non-supplemented cows. The increased blood glucose, insulin and IGF-1 concentrations for supplemented cows indicate an increased energy status, whereas increased concentration of blood NEFA and BHBA for non-supplemented cows suggest a lower energy status (Reist *et al*., 2002). As the concentration of T3 and T4 decreases with stronger negative energy balance (NEB) (Huszenicza *et al*., 2002), non-supplemented cows in current study had lower concentration of T3 and T4 and therefore had a lower energy status compared with supplemented cows. Furthermore, the increased activity of ALAT of non-supplemented cows indicates an increased use of amino acids as an energy source or for gluconeogenesis (Garber *et al*., 1976). The greater activity of ASAT of non-supplemented cows alone should be interpreted with caution. As the activities of GLDH, GGT and CK were not increased for non-supplemented cows, the occurrence of fatty liver syndrome in cows of this study is unlikely.

Results of blood traits indicate that non-supplemented cows were not in strong NEB. Supplementation may not be necessary to balance an organic pasture-based diet, at least for cows in mid-lactation and therefore more severe, but more flexible restrictions over the whole lactation for supplementation are favourable.

Finally, it can be stated that the effects of concentrate supplementation on milk yield, milk composition, grazing behaviour and intake are similar in the present organic study compared with the cited conventional studies.

### Effect of cow strain and consequences for pasture-based organic farming

Another aspect of organic farming is the choice of the breed or strain. This implies the selection of cows adapted to low-input pasture or forage-based feeding system for organic dairy production. In the present study, the milk yield responses obtained for HNZ and HCH cows were similar to the results of Horan *et al*. (2005). Swiss Holstein cows reached almost the overall milk yield response of 1 kg milk/1 kg concentrate as published by Bargo *et al*. (2003), but not in ECM terms. The lower genetic potential for milk production of HNZ cows might be the reason for their lower response of 0.5 kg milk/kg of ingested concentrate, as pasture allowance and mass was the same for both strains. Cows with high genetic potential for milk yield have a greater milk yield response to concentrate supplementation (McCarthy *et al*., 2007b). This phenomenon might be attributed to greater nutrient partition to milk production in high genetic merit cows compared with lower genetic merit cows (Dillon *et al*., 2006). This is supported by the results of IGF-1, with greater plasma concentration in HNZ cows. Greater IGF-1 plasma concentration indicates the coupling of the somatotropic axis, as the liver responds to increased growth hormone concentration and therefore nutrient partitioning favours the build up of body tissue instead of milk production (Lucy *et al*., 2009).

In contrast to results for milk yield, no difference for ECM between cow strains was observed which can be explained by the greater milk protein and in trend greater milk fat content of the HNZ cows. Similarity of ECM yields of the two cow strains indicate a similar efficiency in milk production for HCH and HNZ cows in the present study.

New Zealand Holstein cows ruminated longer than HCH cows, which was also observed in previous studies (Schori and Münger, 2014; Thanner *et al*., 2014). In line with this result HNZ cows tended to spend more time lying down compared with HCH cows as rumination of cows on pasture is associated with lying down (Kilgour, 2012). Furthermore, HNZ cows had a greater number of boli and greater rumination mastication per day compared with HCH cows. Anatomical differences of the muzzle and incisor breadth might explain this (Rook, 2000). Prendiville *et al*. (2010) observed smaller bolus size for the smaller Jersey cows compared with HF cows, indicating that anatomical differences influence the pattern of bolus movement during rumination. Although greater chewing activity during grazing and rumination is associated with a greater salivary secretion and therefore a better fibre digestibility (Domingue *et al*., 1991), HNZ cows could not benefit from longer rumination time in terms of milk production.

The trend for lower pasture DMI by HNZ cows and the lack of clear differences in grazing behaviour do not confirm a greater feeding drive for HNZ cows (McCarthy *et al*., 2007b). It could have been expected that HCH cows had a greater pasture DMI, as DMI and BW are positively correlated (Kertz *et al*., 1991), as BW is usually positively linked to rumen size and therefore intake capacity. Similar pasture DMI indicates a greater DMI per kg BW for HNZ cows compared with HCH cows. Furthermore, HNZ cows spent longer time ruminating. Digestion rate and discharging of the rumen might be increased as rumination determines digestion rate and therefore controls voluntary intake (Bae *et al*., 1983; Gregorini *et al*., 2012).

The substitution rate of pasture DMI is linked to milk response with a lower substitution rate for cows with high genetic potential for milk yield (Bargo *et al*., 2003). However, in the current study, substitution rate, estimated using *n*-alkane, was the same for both cow strains (0.5 kg/kg). The results are in accordance with missing differences for ECM.

Because no differences were observed between cow strains in blood concentration of insulin, NEFA and BHBA, which are indicators of the energy balance of dairy cows (Reist *et al*., 2002), no difference between cow strains are obvious in energy status. However, a trend for an increased concentration of creatinine in blood plasma for HCH cows may indicate a higher skeletal muscle breakdown and the mobilization of more protein as an energy source or for gluconeogenesis. In accordance with results from McCarthy *et al*. (2007a), HNZ cows had greater serum glucose concentration compared with HCH cows. The greater glucose concentration for HNZ cows during breeding season (60 to 150 DIM) represents an important source for energy for the ovary, as Forshell *et al*. (1991) reported an effect of glucose concentration on conception rates. Therefore greater glucose concentration might indicate greater conception rates which is in line with the differences in conception rates reported between HCH cows and HNZ cows (Piccand *et al*., 2013).

The increase in ALAT activity is in accordance with the results of Thanner *et al*. (2014), which might suggest the elevated use of amino acids of the HNZ cows for purposes other than protein synthesis.

Minor differences between cow strains in the current study indicate that both cow strains are equally suited for an organic, pasture-based feeding system. However, the current study was a short-term experiment performed with a small number of animals after the peak of lactation without considering BCS losses, fertility and health traits during a whole lactation, which are important aspects for continuous and successful organic farming. Just choosing a different cow breed or cow strain may not ensure a well-working, low-input organic system. Careful selection of cows that have a high grazing drive ensures the efficient use of pasture for milk production, whereas cows that can deal with lower energy intake at the beginning of lactation and show properties of good health and fertility may be well-suited organic cows.
